# Plant Kinases in the Perception and Signaling Networks Associated With Arthropod Herbivory

**DOI:** 10.3389/fpls.2022.824422

**Published:** 2022-05-04

**Authors:** Gara Romero-Hernandez, Manuel Martinez

**Affiliations:** ^1^Centro de Biotecnología y Genómica de Plantas, Universidad Politécnica de Madrid – Instituto Nacional de Investigación y Tecnología Agraria y Alimentaria, Madrid, Spain; ^2^Departamento de Biotecnología-Biología Vegetal, Escuela Técnica Superior de Ingeniería Agronómica, Alimentaria y de Biosistemas, Universidad Politécnica de Madrid, Madrid, Spain

**Keywords:** Arabidopsis, arthropod herbivore, protein kinases, plant defense, transcriptomics

## Abstract

The success in the response of plants to environmental stressors depends on the regulatory networks that connect plant perception and plant response. In these networks, phosphorylation is a key mechanism to activate or deactivate the proteins involved. Protein kinases are responsible for phosphorylations and play a very relevant role in transmitting the signals. Here, we review the present knowledge on the contribution of protein kinases to herbivore-triggered responses in plants, with a focus on the information related to the regulated kinases accompanying herbivory in Arabidopsis. A meta-analysis of transcriptomic responses revealed the importance of several kinase groups directly involved in the perception of the attacker or typically associated with the transmission of stress-related signals. To highlight the importance of these protein kinase families in the response to arthropod herbivores, a compilation of previous knowledge on their members is offered. When available, this information is compared with previous findings on their role against pathogens. Besides, knowledge of their homologous counterparts in other plant-herbivore interactions is provided. Altogether, these observations resemble the complexity of the kinase-related mechanisms involved in the plant response. Understanding how kinase-based pathways coordinate in response to a specific threat remains a major challenge for future research.

## Introduction

Protein kinases constitute one of the largest gene families in plant genomes. These enzymes catalyze the reversible phosphorylation of specific amino acids (serine, threonine, and tyrosine) to regulate the activity of their target proteins. Protein kinases possess a catalytic domain composed of 250–300 amino acid residues, which was used to classify plant protein kinases into 9 major groups: AGC (Protein Kinase A, G, and C families), CAMK (Calmodulin/Calcium regulated kinases), CK1 (Casein/Cell Kinase 1), CMGC (CDK, MAPK, GSK3 and CLK families), RLK-Pelle (Receptor-Like Kinases), STE (Homologs of the yeast STE genes), TKL (Tyrosine Kinase-Like), Plant-specific, and the Others group (Lehti-Shiu and Shiu, 2012). In the model plant *Arabidopsis thaliana*, about 4% of the genes encode protein kinases, referred to as the Arabidopsis kinome (Champion et al., 2004; Zulawski et al., 2014). More than 60% of all Arabidopsis protein kinases belong to the large group of receptor kinases (RLK-Pelle) (Shiu and Bleecker, 2001, 2003). This group includes transmembrane receptor kinases composed of a variable extracellular domain, a transmembrane segment, and a cytoplasmic domain with kinase activity, as well as receptor-like cytoplasmic kinases (RLCK), which lack the extracellular and transmembrane domains. Among the soluble kinases, the most prominent groups are the CAMK group including kinases related to calcium signals, the CMCG group containing cyclin-dependent kinases (CDK) involved in cell-cycle regulation, and the STE group formed mostly by mitogen-activated protein kinases (MAPKs), involved in signal transmission for responses to extracellular stimuli.

The abundance and variability of protein kinases are directly related to the numerous cellular and biological processes controlled by phosphorylation events. Most of these processes are linked to the responses of the plant to environmental signals. Plants must perceive and respond to many biotic and abiotic stresses to survive, and phosphorylation is one of the major post-translational modifications affecting the activity of regulatory proteins (Chen Y. et al., 2021). Among biotic stresses, most kinases participating in plant defense have been identified in response to plant pathogens. In the last years, considerable efforts have been done to describe the role of RLKs in the perception of pathogen attack as well as the implication of RLCKs, calcium-dependent protein kinases (CDPKs), calcineurin B-like interacting protein kinases (CIPKs), and MAPKs in the transmission of signals (Steinbrenner, 2020; Sun and Zhang, 2020; Escocard de Azevedo Manhães et al., 2021).

In contrast, the participation of phosphorylation signals has been poorly documented in response to herbivory. A number of features common in the plant response to the attack of arthropod herbivores include the depolarization of the membrane, the initiation of intracellular Ca^2+^ spikes, the production of reactive oxygen/nitrogen species, and the triggering of phosphoprotein cascades (Erb and Reymond, 2019). These events lead to the activation of downstream responses to herbivore-specific cues, including transcriptional activation of genes encoding defensive proteins and enzymes for the biosynthesis of toxic compounds (Howe and Jander, 2008; Wu and Baldwin, 2010).

Here, we review the present knowledge on the contribution of protein kinases to herbivore-triggered responses in plants, with a focus on the main groups related to the perception and transmission of signals accompanying herbivory in Arabidopsis.

## Protein Kinases Induced by Arthropod Herbivores

The participation of many proteins in stress responses is directly related to the transcriptional induction of the genes that encode these proteins. Thus, transcriptomic analyses should offer clues on groups and individual protein kinases with a higher implication in the general response to herbivores. The data included in the comparative analysis performed previously (Santamaria et al., 2021) may be used as the starting point to address this question. In this analysis, a set of experiments including datasets of differentially expressed genes in herbivore-infested Arabidopsis plants was selected from transcriptomics databases. The final collection was composed of 28 experiments and included different types of herbivores: lepidopterans, mites, aphids, leafminers, thrips, and hemipteran, and several time points when possible.

**FIGURE 1:**
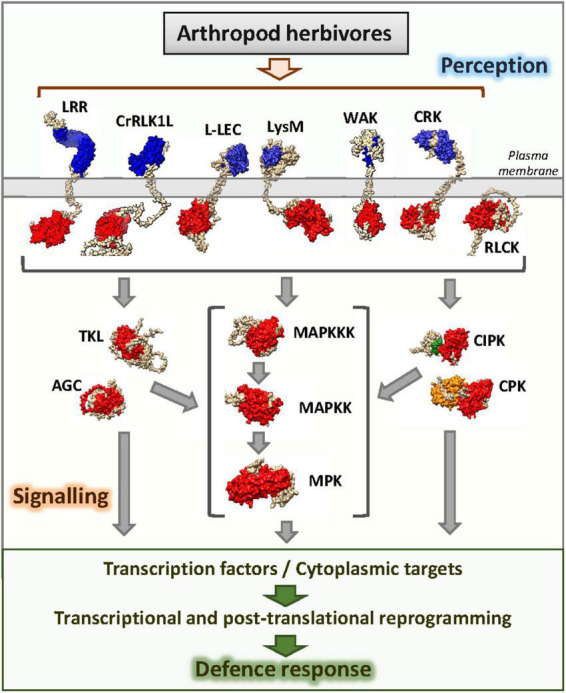
Schematic representation of protein kinase groups participating in the perception and transduction of signals associated with arthropod herbivory. Kinase domains are colored in red, EF-hands are colored in orange, CBL-interacting region is colored in green, extracellular domains involved in the perception of signals associated with herbivory are colored in blue.

After parsing this dataset with the list of Arabidopsis genes encoding protein kinases downloaded from the iTAK database (Zheng et al., 2016) and selecting experiments with more than 20 protein kinases differentially expressed upon herbivore treatment, 63 genes deregulated in at least seven experiments were found (Table 1). These kinases should not be considered universal kinases in response to herbivory since their expression did not change in response to some herbivore treatments. In this review, they are assigned as deregulated by herbivory in the sense that their expression changed in a diverse set of transcriptomic experiments using arthropod herbivores. Six groups of kinases are represented in the list. Members of these groups have previously been involved in different steps related to the perception and transmission of external signals (Figure 1). As expected, the RLK-Pelle group, directly involved in the perception of the attacker, was the most numerous. Likewise, the members of the CAMK and STE groups and the member of the subfamily MAPK of the CMGC group belong to families typically associated with the transmission of stress-related signals. In addition, members of the AGC and TKL groups and one member of the CMGC_CDK subfamily were deregulated.

**TABLE 1:** Differentially expressed kinases in Arabidopsis upon herbivore infestation.

Kinase family	Gene ID	Gene name	Tur_30’	Tur_1h	Tur_3h	Tur_24h	Byo_6h	Byo_2d	Byo_6d	Lhui_4h	Prap_3h	Prap_6h	Prap_12h	Prap_24h	Pbra_2d	Mbra_2d	Splitt_8d	Focc_24h	Focc_48h	Bbra_72h	Mper_24h
**AGC_RSK-2**	**AT3G25250**	**OXI1**	4.38	3.27	3.82	3.13	1.56	0	−2.1	1.98	0	1.58	1.52	2.98	0	0	1.23	0	0	0	2.31
**AGC-Pl**	**AT3G08720**	**ATPK2**	3.91	0	2.46	2.27	0	0	0	1.99	0	0	1.1	1.7	0	0	0	1.4	1.32	0	0
**CAMK_CAMKL-CHK1**	AT5G10930	CIPK5	−1.9	0	0	0	0	0	0	−2	−1.7	0	−1.1	−1.2	−1.8	−2.8	0	0	−3	0	0
**CAMK_CAMKL-CHK1**	AT1G48260	CIPK17	−1.7	−1.3	0	0	−1.1	0	0	0	0	−1.5	−1.7	−1.2	−1.1	0	0	0	0	0	0
**CAMK_CAMKL-CHK1**	AT5G57630	CIPK21	0	0	1.29	1.7	0	1.01	0	4.28	0	0	0	0	2.57	1.02	0	−1	2.45	0	0
**CAMK_CAMKL-CHK1**	AT2G30360	CIPK11	3.21	2.03	2.15	1.63	0	0	0	3.81	2.38	2.42	4.23	2.31	0	0	1.65	0	0	0	0
**CAMK_CDPK**	AT3G19100	CRK2	0	0	0	0	0	0	0	0	1.92	1.58	1.87	2.64	0	1.08	1.28	1.89	0	0	0
**CAMK_CDPK**	AT5G66210	CPK28	3.49	0	1.08	0	0	0	0	1.56	0	0	1.22	1.61	−2	−1.8	0	1.09	1.38	0	0
**CAMK_OST1L**	AT1G78290	SRK2C	0	0	0	0	0	0	0	−2	−1.1	−1.3	0	0	−1.8	−1.4	0	1.95	−1.9	0	0
**CMGC_CDK-CRK7-CDK9**	AT1G33770	–	0	−2	−1	−1.2	0	0	0	0	1.31	−1.7	0	0	0	1.06	0	0	1.11	0	0
**CMGC_MAPK**	AT1G01560	MPK11	5.43	2.51	3.26	3.27	1.78	0	−1.8	2.9	0	0	1.46	1.85	−1.3	0	0	0	1.09	2.92	1.2
**RLK-Pelle_CrRLK1L-1**	AT5G54380	THE1	1.35	0	0	0	0	0	0	−2	2.8	0	0	1.66	0	2.67	0	0	1.46	0	−1
**RLK-Pelle_CrRLK1L-1**	AT5G61350	–	0	1.33	0	0	0	−1.1	−1.2	0	0	0	0	0	−1.2	−1	0	0	−1.3	0	1.42
**RLK-Pelle_DLSV**	AT1G56120	–	0	0	0	0	0	0	0	0	−1.8	−1.7	−1.6	−1.5	−1.7	0	0	0	0	2.27	1.59
**RLK-Pelle_DLSV**	AT4G04540	CRK39	3.95	3.27	4.51	4.48	2.5	3.05	0	0	0	0	0	−1.6	0	0	0	0	0	0	0
**RLK-Pelle_DLSV**	AT4G23200	CRK12	2.72	2.58	3.05	2.61	2.25	2.84	1.73	0	0	0	0	0	−1.5	0	0	0	0	0	0
**RLK-Pelle_DLSV**	AT4G23210	CRK13	1.55	2.76	4.82	4.93	1.86	2.21	0	2.74	0	0	0	0	0	0	0	0	1.67	0	0
**RLK-Pelle_DLSV**	AT4G04510	CRK38	0	0	3.12	4.57	2.17	3.41	1.52	0	0	0	0	−3.2	0	0	0	0	0	0	2.27
**RLK-Pelle_DLSV**	AT4G04490	CRK36	1.29	0	1.28	1.93	1.55	1.32	0	0	0	0	0	−1.2	−1.2	0	0	0	0	2.41	1.11
**RLK-Pelle_DLSV**	AT4G23150	CRK7	2.45	0	2.21	2.91	1.39	1.75	0	0	0	0	0	−2	−1.2	0	0	0	0	3.99	1.55
**RLK-Pelle_DLSV**	AT4G04500	CRK37	0	0	2.2	3.02	1.28	2.5	0	0	0	0	0	−2.8	−1.6	−1.8	0	0	0	3.22	0
**RLK-Pelle_DLSV**	AT4G23140	CRK6	2.16	2.2	1.65	2.19	1.16	1.14	0	0	0	0	0	−2.1	0	0	0	0	1.39	1.81	0
**RLK-Pelle_DLSV**	AT4G23190	CRK11	3.37	1.02	1.34	0	1	0	0	2.69	0	0	0	0	0	0	0	0	1.56	2.01	0
**RLK-Pelle_DLSV**	AT4G23220	CRK14	4.18	3.51	1.87	1.74	1.01	0	0	3.39	0	0	0	0	0	0	0	0	2.74	2.09	0
**RLK-Pelle_DLSV**	AT4G21390	B120	5.57	2.36	3.09	3.3	1.79	1.73	0	3.39	0	0	1.44	2.14	0	2.53	0	1.55	2.27	0	0
**RLK-Pelle_DLSV**	AT1G61610	–	2.83	3.4	4.19	4.72	3.21	2.72	1.3	4.35	2.75	3.38	4.44	2.72	0	2.07	0	1.22	1.6	0	0
**RLK-Pelle_DSLV**	AT4G11890	–	3.83	3.52	1.63	2.62	1.26	0	−1.1	3.24	0	0	0	−2.2	−1.3	−1.6	0	0	2.71	3.1	0
**RLK-Pelle_L-LEC**	AT5G60300	LECRK19	0	0	1.38	1.46	1.54	1.59	0	2.31	1.33	1.64	2.35	1.94	0	0	2.35	0	0	0	0
**RLK-Pelle_L-LEC**	AT5G65600	LECRK92	2.73	0	1.43	2.8	2.42	2.31	1.51	2.28	0	0	−1.1	0	1.99	2.6	0	0	0	0	1.29
**RLK-Pelle_L-LEC**	AT5G01540	LECRK62	3.58	2.9	2.92	2.64	0	0	0	2.47	0	0	0	0	−2.1	0	0	1.34	0	2.27	0
**RLK-Pelle_L-LEC**	AT1G70130	LECRK52	6.52	7.69	8.92	7.91	0	0	0	0	3.04	3.33	4.57	3.31	0	0	0	0	0	0	2
**RLK-Pelle_LRK10L-2**	AT1G67000	LRK10L-2.8	0	0	1.15	2.18	2.13	2.19	1.76	1.86	0	0	1.07	0	0	0	0	0	0	0	1.17
**RLK-Pelle_LRR-I-1**	AT2G19190	SIRK	0	0	0	1.61	1.68	0	0	2.98	0	0	0	−2.9	−1.7	−1.5	0	0	0	3.02	0
**RLK-Pelle_LRR-I-1**	AT1G51890	–	0	0	0	1.87	1.51	1.79	0	2.71	0	0	0	−2.3	0	0	1.1	0	0	2.57	0
**RLK-Pelle_LRR-I-1**	AT1G51790	–	2.21	1.42	0	1.62	1.26	0	0	1.55	0	0	−1	0	0	0	0	0	1.05	0	0
**RLK-Pelle_LRR-I-1**	AT1G51820	–	3.6	2.38	1.52	1.12	1.59	0	−2.1	2.17	0	2.44	0	1.34	0	0	0	0	0	0	0
**RLK-Pelle_LRR-I-1**	AT1G51800	IOS1	4.25	2.95	1.95	2.41	2.07	1.61	−1.1	3.01	0	0	0	−1.1	0	0	0	0	1.41	0	0
**RLK-Pelle_LRR-Xb-2**	AT1G74360	–	2.78	1.33	1.85	1.9	0	0	0	2.62	0	0	0	0	0	0	0	0	1.35	1.53	0
**RLK-Pelle_LRR-XI-1**	AT1G73080	PEPR1	2.16	0	1.18	0	0	0	0	1.84	1.4	2.02	2.19	2.36	0	0	1.08	0	0	0	0
**RLK-Pelle_LRR-XI-1**	AT5G25930	–	3.27	1.77	0	1.75	1.61	0	0	2.86	0	0	0	0	0	1.52	0	0	2.44	0	0
**RLK-Pelle_LRR-XI-1**	AT1G09970	LRR XI-23	2.87	2.16	2.38	2.07	1.12	1.17	0	2.31	1.24	0	0	1.23	1.49	1.69	2.35	0	2.16	1.93	0
**RLK-Pelle_LRR-XI-1**	AT1G17750	PEPR2	2.27	1.42	2.38	1.62	2.4	1.57	0	2.23	1.66	2.58	3.53	3.24	0	1.25	1.04	0	0	0	0
**RLK-Pelle_LysM**	AT2G33580	LYK5	2.58	1.07	1.57	1.23	0	1.03	0	0	0	0	1.18	1.27	−1.8	0	0	0	0	1.54	1.01
**RLK-Pelle_RLCK-VIIa-2**	AT3G09830	–	3.26	1.55	1.75	1.57	1.2	0	0	2.27	0	0	0	0	0	0	0	0	1.02	0	0
**RLK-Pelle_RLCK-VIIa-2**	AT5G25440	–	2.97	2.26	1.9	1.6	0	0	0	2.43	0	0	0	0	0	0	0	0	1.52	1.6	0
**RLK-Pelle_RLCK-VIIa-2**	AT1G14370	PBL2	2.02	0	1.27	1.29	0	1.33	0	2.17	0	0	0	0	0	0	1.01	0	1.11	1.8	0
**RLK-Pelle_RLCK-VIIa-2**	AT1G69790	PBL18	1.61	1.92	1.65	1.22	0	0	0	1.53	0	0	1.35	0	−1.4	0	0	0	0	0	0
**RLK-Pelle_RLCK-VIII**	AT3G59350	–	3.88	0	0	0	0	0	0	2.03	1.9	0	0	1.2	1.91	2.02	0	0	3.75	0	0
**RLK-Pelle_RLCK-XIII**	AT4G10390	–	2.28	3.06	3.45	2.78	3.7	2.83	0	3.07	2.66	3.47	4.13	2.27	1.36	2.25	3.68	1.75	0	0	0
**RLK-Pelle_SD-2b**	AT5G24080	–	3.4	4.12	3.22	2.51	0	0	0	3.48	4.81	4.13	5.79	6.27	1.08	0	0	0	0	0	−1
**RLK-Pelle_WAK**	AT4G31110	WAKL18	1.61	1.25	1.64	2.98	0	0	0	0	0	1.96	3.05	3.4	0	0	0	0	0	0	0
**RLK-Pelle_WAK**	AT1G79680	WAKL10	3.94	0	2.5	3.92	3.86	0	0	4.35	0	0	1.23	0	0	2.52	0	0	2.42	1.91	1.36
**STE_STE11**	AT2G32510	MAPKKK17	1.1	1.78	3	2.08	3.21	3.32	0	2.44	2.35	3.01	4.17	2.35	0	2.03	1.88	0	0	0	0
**STE_STE11**	AT5G67080	MAPKKK19	3.25	3.1	3.59	3.66	5.56	3.17	0	4.58	3.42	3.76	4.85	1.43	1.68	2.52	0	2.65	1.79	1.7	0
**STE_STE11**	AT1G05100	MAPKKK18	2.86	1.74	2.29	0	0	0	0	3.51	3.45	3.03	4.48	4.13	0	0	0	0	0	0	0
**STE_STE11**	AT2G30040	MAPKKK14	2.55	0	2.41	1.41	0	0	0	2.23	1.59	3.17	3.73	2.9	−2.1	0	1.33	0	0	0	1.62
**STE_STE11**	AT4G36950	MAPKKK21	6.29	6.99	7.19	5.89	4.96	2.37	0	0	2.73	3.24	4.47	1.31	0	0	0	0	0	0	0
**STE_STE7**	AT1G73500	MKK9	2.13	1.31	0	1.57	0	1.39	0	2.53	0	1.28	1.01	1.25	0	0	1.76	1.12	1.01	1.68	0
**TKL_CTR1-DRK-1**	AT2G31010	–	1.92	0	0	0	0	0	0	0	2.05	0	0	0	1.8	2.32	0	−1.3	1.83	0	−1.4
**TKL-Pl-4**	AT5G40540	–	2.87	1.44	1.55	0	0	0	0	0	1.15	2.6	1.87	1.73	0	0	0	0	0	0	0
**TKL-Pl-4**	AT5G01850	–	0	1.68	2.63	1.71	1.36	0	0	0	0	1.09	1.79	0	0	0	1.93	0	0	0	0
**TKL-Pl-4**	AT4G38470	STY46	0	0	0	0	0	0	0	1.55	1.86	0	0	1.21	1.79	1.67	0	0	1.11	0	−1.1
**TKL-Pl-5**	AT4G18950	–	2.8	0	1.08	0	0	0	0	0	1.03	1.26	1.82	1.37	0	0	0	0	1	0	0

*Numbers refer to the log2FC values exhibited in herbivore-infested plants compared to control plants. Herbivore and time of infestation are included for each individual experiment. Data were extracted from the dataset previously used in Santamaria et al. (2021). Tur, Tetranychus urticae; Byo, Brevipalpus yothersi; Lhui, Liriomyza huidobrensis; Prap, Pieris rapae; Pbra, Pieris brassicae; Mbra, Mamestra brassicae; Splitt, Spodoptera littoralis; Focc, Frankliniella occidentalis; Bbra, Brevicoryne brassicae; Mper, Myzus persicae. Background red/green pattern corresponds to up/down regulated genes.*

To highlight the importance of these protein kinase families in the response to arthropod herbivores, a compilation of knowledge on their members will be provided in the following sections. If available, a thorough description of their functionality against herbivores will be done, which will be compared with previous findings on their role against pathogens. Finally, the information of their homologous counterparts in other plant-herbivore interactions will be provided.

## RLK-Pelle Group

The RLK-Pelle group is composed of proteins with a cytoplasmic kinase domain involved in the early signaling steps upon the perception of extracellular stimuli. This group is formed by kinases with and without extracellular domains. Based on the ectodomains, Arabidopsis transmembrane RLKs can be classified into 14 types (Jose et al., 2020). Many of these types are represented in the most upregulated genes upon herbivory (Table 1). According to their higher abundance in the Arabidopsis genome, many herbivore-regulated kinases belong to the leucine-rich repeat (LRR) subgroup or to the DLSV/DSLV subgroup, formed by kinases with the Domain of Unknown Function 26 (DUF26), also called Cysteine-rich Receptor-like Kinases (CRKs). In addition, members of some other subgroups are included in the Table, such as the L-lectin (L-LEC), the wall-associated (WAK), the *Catharanthus roseus* RLK1-like (CrRLK1L), the S domain 2b (SD-2b), the LysM domain-containing (LysM), and the LRK10-like type 2 (LRK10L-2).

In these proteins, the kinase activity depends on the interaction of the extracellular domain with its specific interactors. Typically, compounds present in pathogens and pests are recognized by these domains. Among the best-characterized RLKs are the LRR-type receptors FLS2 (flagellin-sensitive 2) and EFR (elongation factor Tu receptor), which recognize universal bacterial proteins (Kunze et al., 2004; Chinchilla et al., 2007). Likewise, the LysM-type RLKs LYK5 (lysine motif receptor kinase 5) and CERK1 (chitin elicitor receptor kinase 1) bind bacterial and fungal carbohydrates (Miya et al., 2007; Cao et al., 2014). Despite the crucial role of the receptors in the perception of elicitors associated with herbivory, little is known on the particular RLKs involved in these interactions (Reymond, 2021).

A detailed analysis of the most common RLKs induced by herbivores provides interesting insights into the general mechanisms triggered in the plant. The most relevant finding is the shared induction by pathogens and pests of a set of RLKs in response to damage or danger-associated molecular patterns (DAMPs). These receptors are involved in the recognition of intracellular and cell wall derived molecules released upon damage and in the reception of induced secreted peptides or phytocytokines produced in the attacked cell to alert other cells (Li P. et al., 2020; Zhou and Zhang, 2020). Upon cell damage, plant cells secrete peptides that are preferentially recognized by members of the LRR subgroup. Among the scarce peptide-receptor pairs previously described, some receptors of the RLK-Pelle_LRR-XI-1 group are present in the herbivore-regulated dataset. PEPR1 and PEPR2 sense secreted Peps (plant elicitor peptides) and LRR XI-23 (RLK7) binds PIPs (PAMP-induced secreted peptides), inducing the reinforcement of immune signaling (Huffaker et al., 2006; Hou et al., 2014).

In contrast, there are several classes of RLKs capable of sensing cell wall derived carbohydrate ligands, including receptors from the WAK, LysM, CrRLK1L, and LRR families (Bacete et al., 2018; Lorrai and Ferrari, 2021). Several WAK proteins have been reported to bind cell wall-associated pectins or wound-induced short oligogalacturonic acid fragments derived from pectin (Kohorn et al., 2009; Brutus et al., 2010). The herbivore-responsive protein THE1 is a member of the CrRLK1L family that has been suggested to participate in the perception of unknown cell wall damage-derived signals (Hématy et al., 2007). The LRR-RLK MIK2 has been also proposed to carry out a role in sensing cell wall damage, probably functioning in the same pathway as THE1 (Van der Does et al., 2017). Finally, several members of the LysM containing family have been broadly involved in recognizing saccharide elicitors. For effective chitin-triggered immune signaling, the LysM receptors CERK1 and LYK5 are necessary (Cao et al., 2014). Broadly deregulated upon herbivore attack, LYK5 protein binds chitin with higher affinity than CERK1, but their kinase domain is inactive. In this manner, CERK1-LYK5 heterodimers are required for the activation of immune responses (Cao et al., 2014). Moreover, CERK1 has been shown to be necessary for immune responses triggered by 1,3-b-D-glucans present in plant cell walls (Mélida et al., 2018). Likewise, mixed-linked β-1,3/1,4-glucans present in some plant cell walls, such as the trisaccharide β-D-cellobiosyl-(1,3)-β-D-glucose, trigger immune responses partially dependent on the LysM members CERK1 and LYK5 (Rebaque et al., 2021).

Interestingly, other families could act as carbohydrate-binding receptors. Several families present an extracellular lectin or lectin-like domain, such as the L-LEC and SD-2b families (Bellande et al., 2017). In addition, the cysteine-rich receptors of the DLSV family have two extracellular lectin-like domain folds (Vaattovaara et al., 2019), probably binding chitin oligomers (Vanholme et al., 2014). However, members of these families are also able to sense non-carbohydrate ligands. The L-LEC receptors LECRK15 and LECRK19 (DORN1) binds extracellular ATP (eATP), which is assumed to be released during cell damage (Choi et al., 2014; Pham et al., 2020). LECRK62 and LECRK18 are potential receptors for extracellular NAD^+^ (eNAD^+^) and NADP^+^ (eNADP^+^) and play pivotal roles in plant immunity (Wang et al., 2017, 2019). Again, the danger sensors *LECRK19* and *LECRK62* are present in the herbivore-regulated dataset. In addition, two other L-LEC receptors, *LECRK92* and *LECRK52*, are broadly deregulated upon herbivore attack. The kinase domain of LECRK92 recruits calcium-dependent protein kinases to phosphorylate RBOHD for immune activation (Luo et al., 2017; Xu et al., 2020). LECRK52, together with LECRK71, is critical in MeJA-mediated stomatal closure in response to the bacterial attack (Yekondi et al., 2018). Although the ligands for SD-2b and CRK families are still unknown, their implications in plant immunity have been largely documented. The herbivore-responsive SD-2b receptors have been previously related to plant defense. *At1g61610* has been shown to be upregulated by flagellin, chitosan, and *P. syringae* DC3000 (Ko et al., 2006; New et al., 2015). *At4g21390* is upregulated in the presence of fungal elicitors (Chae et al., 2009). Likewise, most of the CRK receptors widely deregulated by herbivores have been formerly associated with disease resistance and cell death in plants. In gene expression analyses, most CRKs were induced by pathogen treatment or biotic-related compounds (Wrzaczek et al., 2010; Bourdais et al., 2015; Lee et al., 2017). Besides, overexpression of *CRK6*, *CRK13*, *CRK36*, and *CRK45* in Arabidopsis led to enhanced resistance to *P. syringae* DC3000 (Chen et al., 2003; Acharya et al., 2007; Zhang et al., 2013; Yeh et al., 2015).

As above stated, little is known about the functional characterization of RLKs in response to herbivory and the consequences of their transcriptional deregulation on the triggered signaling pathways. Regarding Arabidopsis responses to herbivory, very scarce information has been provided. The Arabidopsis LRR-RLK BAK1 is required for the defense response to aphid attack (Prince et al., 2014). The critical function of BAK1 as a co-receptor for RLKs and RLPs (Receptor-Like Proteins, similar to RLKs but lacking the kinase domain) supports its relevance in sensing herbivory (DeFalco and Zipfel, 2021). Besides, the oral secretion extracted from larvae of *Spodoptera litura* caused the formation of homomultimers of an LRR-RLK, AtHAK1, that interacted with the cytoplasmic signaling kinase PBL27 resulting in herbivory resistance in an ethylene-dependent manner (Uemura et al., 2020). Finally, an L-type *LecRLK18* was found to be locally upregulated upon *Pieris brassicae* oviposition and egg extract treatment. In *lecRK18* mutant plants, egg extract treatment caused a significant reduction of ROS, SA production, *PR1* expression, and cell death (Gouhier-Darimont et al., 2019). Recent evidences suggest that LecRLK11 functions in the same signaling pathway as LecRLK18 in response to eggs of *P. brassicae* (Groux et al., 2021).

In addition, several findings have been reported for the perception of herbivores by RLKs in other plant-herbivore models. The rice leucine-rich repeat receptor-like kinase 1 (OsLRR-RLK1) is involved in the defense responses of rice against striped stem borer (Hu et al., 2018). Besides, two RLKs from soybean, GmHAK1 and GmHAK2, are herbivore-specific RLKs mediating herbivore danger transmission caused by *S. litura* (Uemura et al., 2020). Similar to the involvement in plant-microbe interactions, it has been shown in several plant species that LecRLKs are involved in the perception of insect feeding. A G-type LecRLK in *Nicotiana attenuata*, *NaLecRK1*, was transcriptionally induced under the attack of *Manduca sexta*, which promotes the suppression of SA production and contributes to the accumulation of JA-mediated defense response (Gilardoni et al., 2011). Besides, rice LecRKs were shown to serve positively for defense responses of the host plant during damage by the herbivore *Nilaparvata lugens* (Liu et al., 2015; Ye et al., 2020). More recently, a cowpea LRR-RLP was described as a receptor of inceptins, molecules responsible for elicitor-induced responses and enhanced defense against *Spodoptera exigua* (Steinbrenner et al., 2020).

## Calcium-Regulated Kinases

In plants, the calcium ion (Ca^2+^) is a second messenger involved in the regulation of diverse physiological responses. In basal conditions, calcium concentrations are relatively low, around 10^–7^ M in the cytosol. However, upon perception of a variety of stimuli, the calcium content of the cytosol rapidly increases, with rising concentrations around 10^–6^ M (McAinsh and Pittman, 2009; Dodd et al., 2010). To detect the increase and influx of calcium to the cell, several sensors play a relevant role. These sensors typically present a motif Elongation Factor-hand (EF-hand) to bind free Ca^2+^ or to regulate the ion homeostasis acting as Ca^2+^ chelators (Mohanta et al., 2019). When calcium is sensed, a conformational change is produced to let downstream signals, commonly related to the activation of calcium-regulated protein kinases (Sanyal et al., 2016). Four major groups of Ca^2+^ decoders play a role in this process: Calmodulins (CaMs), Calcineurin B-Like proteins (CBLs), Calcium-Dependent Protein Kinases (CDPKs or CPKs), and CDPK-Related Kinases (CRKs). CBLs bind calcium and trigger phosphorylation signaling by its interaction with CBL-Interacting Protein Kinases (CIPKs), a group of kinases specific for plants (Ma et al., 2020). CPKs and CRKs are two closely related families of kinases. While CPKs harbor functional EF-hands, degenerate EF-hands are found in CRKs. Contribution to calcium signaling by CRKs could be associated with their capacity to interact with calcium-binding CaMs (Yip Delormel and Boudsocq, 2019). Regarding the role in plant defense of calcium-regulated protein kinases, it has been largely reported that CIPKs and CPKs are needed to sense and decode Ca^2+^ signaling induced by pathogens into phosphorylation events (Seybold et al., 2014; Yuan et al., 2017; Bredow and Monaghan, 2019). However, little is known on the role of particular Ca^2+^ sensors in response to herbivory.

Seven calcium-regulated kinases were found as associated with herbivory (Table 1). Four of them are CIPKs. CBL-CIPKs pairs have been associated with the regulation mediated by reactive oxygen species (ROS), which are generated as a response to the attack of herbivores or pathogens and regulate intracellular calcium concentration (Ma et al., 2020). ROS function as signaling molecules in response to biotic stresses and are closely linked to RBOH (respiratory burst oxidase homolog) proteins. RBOHF together with RBOHD functions in ROS-dependent ABA signaling in guard cells and both are required for ROS signaling in plant defense responses (Marino et al., 2012). As RBOHF is regulated by CIPK11 (Han et al., 2019), the upregulation of *CIPK11* by herbivory would trigger the activation of RBOHF and the release of Ca^2+^. Besides, many CBL-CIPK pairs have been related to the regulation of ion channels both in the plasma membrane and the tonoplast (Saito and Uozumi, 2020). Interestingly, two herbivore-responsive CIPKs (*CIPK5* and *CIPK17*) were commonly downregulated, and the other two (*CIPK11* and *CIPK21*) upregulated. CIPK11 has been associated with movement to ensure rapid stomatal closure, which is a widely reported response to different abiotic and biotic stresses. CIPK11 attenuates stomatal opening by phosphorylation of the plasma membrane H + -ATPase AHA2 (Fuglsang et al., 2007; Yang et al., 2010) and promotes stomatal closing by activation of the anion channel SLAC1 (Geiger et al., 2010). In addition, CIPK11 phosphorylates and activates ABI5, a transcription factor involved in ABA signaling (Zhou et al., 2015). ABI5 has been associated with a positive function in plant ABA signaling and thus, with processes leading to acquire tolerance to abiotic stresses, such as stomatal regulation (Collin et al., 2021). Likewise, CIPK5 phosphorylates and activates the potassium efflux channel GORK, inducing stomatal closure (Förster et al., 2019). This process is triggered by wounding through the JA signaling cascade. Strikingly, despite the shared role of CIPK11 and CIPK5 in stomatal closure, herbivory commonly induces the expression of *CIPK11* and represses the expression of *CIPK5*. In contrast, *CIPK17* and *CIPK21* showed a consistent pattern to that observed for *CIPK11*. *CIPK17* was downregulated by herbivory and has been proposed to negatively regulate ABA signaling during stomatal movement (Song et al., 2018). *CIPK21* is upregulated by herbivory and ABA treatment and mediates the response to salt and osmotic stress in the tonoplast (Pandey et al., 2015).

On the other hand, two kinases of the CAMK-CDPK group, *CPK28* and *CRK2*, were typically upregulated by herbivory. CPK28 has a negative effect in plant defense against pathogens and has not been previously associated with responses to herbivores. This kinase negatively regulates PAMP-induced ROS signaling by reducing the stability of the RBOHD stimulating enzyme BIK1 through the phosphorylation and activation of the plant U-box type E3 ubiquitin ligases PUB25/26 (Monaghan et al., 2014; Wang et al., 2018). In contrast, CRK2 has been implicated in the defense response of Arabidopsis plants against the generalist herbivore *S. litura* (Miyamoto et al., 2019). CRK2 phosphorylates and activates the ethylene-responsive transcription factors ERF13 and RAP2.6 (Nemoto et al., 2015), and was positively regulated by JA and ABA. Besides, Arabidopsis plants overexpressing CRK2 or CRK3 showed increased expression levels of the defensin gene *PDF1.2*, and a higher protection against *S. litura* (Miyamoto et al., 2019). In a previous report, the importance of the CPK3 and CPK13 proteins was determined in Arabidopsis plants attacked by *Spodoptera littoralis* (Kanchiswamy et al., 2010). Following attack, the *cpk3* and *cpk13* mutants showed reduced transcript levels of *PDF1.2*. These results support a role of members of both CPK/CDPK and CRK groups in the response of the Arabidopsis plant to herbivory.

Finally, *SRK2C*/*SnRK2.8* was the only member of the CAMK_OST1L family deregulated by herbivory. This kinase is involved in plant defense against pathogens. SRK2C/SnRK2.8 is induced by SA-independent systemic signals and phosphorylates the transcription factor NPR1 during systemic acquired response. This phosphorylation is necessary for the import of NPR1 to the nucleus, where it induces the expression of defensive pathogenesis-related genes (Lee et al., 2015). *SRK2C*/*SnRK2.8* was commonly downregulated by herbivore attack, which highlights the global dissimilarity in the plant response to pathogens and herbivores.

## Map Kinases

Mitogen-Activated Protein Kinases (MAPKs) are conserved signaling proteins present in all eukaryotes. In plants, MAPKs have been related to development, abiotic, and biotic factors (Zhang et al., 2018). They are characterized by the formation of cascades of three different kinases activated by phosphorylation (Krysan and Colcombet, 2018). The first kinase involved in the cascade is a MAPKKK (also called MAP3K or MEKK), triggered by external factors, sensors, or receptors (as RLKs/RLPs). Once a MAP3K is activated, it can phosphorylate Ser/Thr amino acids in the activation loop of the second protein of the cascade, a MAPKK (also named MAP2K, MKK, or MEK). Finally, a MAPK (or MPK) is phosphorylated by the previous one in Thr/Tyr amino acids located in the activation loop (Jagodzik et al., 2018). The activation of the last MAPK of the cascade triggers the regulation by phosphorylation of other kinases, transcription factors, or enzymes. In *A. thaliana* has been found 80 MAPKKKs, 10 MAPKKs, and 20 MAPKs (Colcombet and Hirt, 2008). Five MAP3Ks, *MAPKKK14*, *17*, *18*, *19*, and *21*; the MAP2K *MKK9*; and the MAPK *MPK11* were commonly regulated by herbivory (Table 1).

The relationship between MAPK cascades and defense has been largely reported (Thulasi Devendrakumar et al., 2018; Zhang et al., 2018). In Arabidopsis, most reports have been focused on the activated MAPK cascades in response to pathogens. These cascades typically involve the MAPKs MPK3, MPK4, and MPK6, controlled by less characterized MAP3K/MAP2K modules. MPK3/6 perform redundant functions upon *Botrytis cinerea* or flagellin treatment, which include the activation of the transcription factors WRKY33 and ERF104, and the induction of genes involved in the synthesis of camalexin and ethylene (Bethke et al., 2009; Mao et al., 2011; Li et al., 2012). The wound-induced activation of *MPK3* and *MPK6* is enhanced by grasshopper oral secretions (Schäfer et al., 2011). Besides, MPK4 is required for basal defense (Zhang et al., 2012). Despite this general stress-responsive role for MPK3/4/6 kinases, any of them was consistently deregulated in response to herbivores. The only MAPK induced by herbivory is *MPK11* (Table 1). *MPK11* is also activated in response to flg22, a 22 amino acid PAMP derived from bacterial flagellin, and responds to elf18 (derived from bacterial elongation factor EF-Tu) and ch8 (N-acetylchitooctaose derived from fungal chitin). Both PAMPs led to rapid *MPK11* transcript accumulation and increased MPK11 kinase activity (Bethke et al., 2012; Eschen-Lippold et al., 2012). In addition, MPK11 and MPK4 interact and phosphorylate the transcription factor ERF8 *in vitro*. ERF8 has been involved in resistance to the bacterial pathogen *P. syringae* (Cao et al., 2018). Again, the role of MPK11 in plant-herbivore interactions remains unknown.

MPKs are the final step of the MAP kinase cascade. To date, four cascades have been associated with biotic stresses in Arabidopsis, MAPKKK3/5/MEKK1-MKK4/5-MPK3/6; MEKK1-MKK1/2-MPK4; MAPKKK14-MKK3-MPK1/2/7; MAPKKK?-MKK9-MPK3/6 (Krysan and Colcombet, 2018; Lin et al., 2021). Among the participants in these cascades, *MKK9* and *MAPKKK14* were found upregulated by herbivory (Table 1). MKK9 is an upstream activator of MPK3 and MPK6 both *in vitro* and *in planta*. Overexpressing *MKK9* plants induced the synthesis of ethylene and camalexin through the activation of MPK3 and MPK6 (Xu et al., 2008). To date, a direct participation of MKK9 in response to herbivores has not been reported. In the case of *MAPKKK14*, its expression was induced in the response to JA produced after wounding treatment (Sözen et al., 2020). Wound-induced MAPKKK14 activates the MKK3-MPK1/2/7 module in a MKK4/5-independent manner. Notably, *mkk3* mutant plants were more susceptible to herbivory from larvae of *S. littoralis*, supporting a role of the MAPKKK14-MKK3-MPK1/2/7 module in counteracting insect feeding. Among the rest of MAP3K induced by herbivory, *MAPKKK17*, *18*, and *19* were also induced by wounding (Sözen et al., 2020), which could be associated with their participation in similar herbivore-related modules. In addition, *MAPKKK17* and *18* are induced by ABA (Danquah et al., 2015). MAPKKK17/18 activates the MKK3-MPK1/2/7/14 module, and the entire MAPKKK17/18-MKK3-MPK1/2/7/14 module is regulated by ABA. Interestingly, mutant *mapkkk18* plants are compromised to close stomata in response to ABA (Mitula et al., 2015), which could be related to a role in plant defense as open stomata make the plants more vulnerable to microbial invasion.

Further findings have been reported on the relevance of MAP kinase cascades in plant responses to herbivory in other plant-herbivore models (Hettenhausen et al., 2015). In *N. attenuata* plants, mechanical wounding and oral secretion of the herbivore *M. sexta* activate the MAP kinases SIPK and WIPK (Wu et al., 2007). These kinases regulate the levels of JA and SA in wounded tobacco plants (Seo et al., 2007). In tomato, *M. sexta* feeding activated the WIPK and SIPK homologs SlMPK3 and SlMPK1/2. In silencing *SLMPK1/2* plants, *M. sexta* larvae grew better and caused a lower increase of JA levels (Kandoth et al., 2007). Regarding the MAP kinase MPK4, while silencing *MPK4* in tobacco plants compromises the induction of JA-responsive genes (Gomi et al., 2005), silencing *N. attenuata MPK4* plants did not affect JA production in response to the oral secretion of *S. littoralis* but negatively affected the induction of JA levels by oral secretions of *M. sexta* (Hettenhausen et al., 2013). In rice, several OsMAPKs modulate herbivory-induced phytohormone signaling pathways (Chen J. et al., 2021). OsMAPKK3 regulates hormone dynamics in the interaction between rice and the planthopper *N. lugens* (Zhou et al., 2019). OsMAPK3 and OsMAPK4 act as positive regulators conferring resistance to the lepidopteran *Chilo suppressalis*. OsMAPK3 modulates JA signaling pathway and promotes the accumulation of trypsin protease inhibitors (Wang et al., 2013). OsMAPK4 modulates JA, ET, and SA signaling pathways (Liu et al., 2018). Additionally, OsMAPK20-5 negatively regulates the accumulation of ET and NO, and rice resistance to *N. lugens* (Li et al., 2019).

## Other Kinases. TKL and AGC Groups

Members of two additional groups of kinases (TKL and AGC) are also shown in Table 1. Five genes belong to the TKL group and two genes to the AGC group. The TKL group is formed by dual serine/threonine and tyrosine kinases, also named STY kinases. The AGC group is a heterogeneous group of serine/threonine kinases that does not include the AGC-PI subgroup, which is located in the others group.

The TKL group has not been extensively studied, but some members could be involved in the regulation of some plant metabolic and developmental processes. One of the genes differentially expressed upon herbivory, *STY46*, encodes a protein associated with the phosphorylation of transit peptides of chloroplast and mitochondria-targeted proteins (Lamberti et al., 2011; Law et al., 2018). STY46 has also been involved in the regulation of lipid metabolism and the response to abiotic stresses (Parthibane et al., 2012; Dong et al., 2020). Regarding plant defense, the induction of its expression by MeJA and ethylene suggests a role for *STY46* (Rudrabhatla et al., 2006). Likewise, herbivore-induced *STY5* and *STY12* were upregulated by MeJA or SA treatments (Rudrabhatla et al., 2006). However, a direct association with the response of the plant to pathogens or herbivores has not been reported for any protein of this unexplored group yet.

Contrarily, the role of several members of the AGC group has been thoroughly characterized. These proteins play essential roles in many physiological processes related to cell growth and differentiation, and in response to different stresses (Hirt et al., 2011). One of the herbivore-induced kinases is *OXI1*, required for ROS-mediated responses and for immunity against the oomycete *Hyaloperonospora arabidopsidis* and the bacteria *P. syringae* DC3000 (Rentel et al., 2004; Petersen et al., 2009). OXI1 is also required for the full activation of MAPK3 and MAPK6 in response to cellular injury and oxidative stress (Rentel et al., 2004). Jasmonate-related responses are linked to OXI1. The *oxi1* mutant showed a reduced accumulation of jasmonate together with a downregulation of genes associated with the JA response, whereas genes that respond to SA were predominantly upregulated (Shumbe et al., 2016). Interestingly, silencing *oxi1* mutants were more resistant to the aphid *M. persicae*, which is in agreement with the relevance of the SA-induced response triggered by this aphid (Shoala et al., 2018). The second herbivore-induced kinase is *AtS6K2*/*AtPK2*, which has been mainly related to plant growth and exerts a positive regulation of ABA response and drought resistance (Li L. et al., 2020). Besides, AtS6K2 interacts with the viral genome-linked protein of Turnip mosaic virus and Potato virus A, and this interaction has the potential to interfere with AtS6K2 function (Rajamäki et al., 2017). Thus, AtS6K2 could play an important role in both biotic and abiotic stresses.

## Discussion

The participation of kinases in signaling is unquestionable. However, unraveling the role of each individual kinase represents a major challenge. Existing available datasets derived from high-throughput experiments have been revealed as robust tools to obtain valuable information. Regarding kinases, transcriptomics and proteomics analyses are essential to understanding the role of kinases in perception and response to herbivore attacks. As post-translational modifications are common regulatory forms for many stress-related proteins, phosphoproteomic analyses can determine the relevance of the phosphorylation events caused by herbivore-induced kinases. However, searches in relevant proteomics databases, such are the ProteomeXchange consortium of proteomics resources^1^ and the Arabidopsis Protein Phosphorylation Site Database (PhosPhAt 4.0^2^), reported a lack of proteomic datasets in the Arabidopsis response to herbivory. Whereas proteomics analyses have been done in response to pathogens, elicitors, or hormonal treatments, any experiment using herbivory is available in the databases yet. In contrast, the transcriptomics responses of Arabidopsis plants to many herbivore threats have been reported. In this way, parsing transcriptomic data included in a previous comparative analysis using Arabidopsis as a plant model permitted us to obtain several clues on the kinase machinery involved in plant response to arthropod herbivores.

The first clue is the participation of many kinase groups in the signaling pathways. As expected, phosphorylation events in the response of the plant to the herbivore mainly fall on kinases involved in the perception of exogenous signals and endogenous calcium, as well as in kinases participating in the transmission of the signal to the nucleus. Notably, most Arabidopsis transmembrane RLK types are represented in the most deregulated genes upon herbivory, including members of the LRR, DLSV/DSLV, L-LEC, WAK, CrRLK1L, SD-2b, LysM, and LRK10L-2 groups. Likewise, calcium-regulated kinases from the CIPK, CDPK, and OST1L groups are deregulated by herbivory, as well as members involved in the three levels of MAP kinase cascades, MAP3K, MAP2K, and MPK. Altogether, these observations resemble the complexity of the response.

The second point focuses on the particularities of the kinases involved in the perception mechanisms. The final response to an external stimulus is not linear, it depends on a network of protein interactions with intrinsic redundancies. Multiple receptors and transmitters are activated to confront a threat, which commonly is perceived as a combination of stimuli. Herbivore responses displayed a general behavior fitting this multiple perception mechanism. Typical damage or danger-associated transmembrane receptors are commonly activated by herbivory. These receptors are involved in the recognition of cell wall derived molecules released upon damage and in the reception of induced secreted peptides produced in the attacked cell. Besides, multiple RLKs capable of sensing carbohydrate ligands are upregulated, probably binding chitin oligomers but also able to sense non-carbohydrate ligands. In addition, oral secretions, pheromones, vibrations, pest symbionts, eggs, and frass could contribute to the perception of an herbivore threat. In most aspects, whether these stimuli trigger general or specific mechanisms remains to be elucidated.

The third tip affects the particularities in the specific response to herbivory. Several herbivore-associated Arabidopsis kinases have not been previously described in response to pathogens. Although we cannot discard an unidentified relevant role in the response to microbial pathogens, these kinases could be specifically involved in the perception of the plant to a common signal associated with herbivory.

Understanding how kinase-related mechanisms coordinate in response to a specific threat remains a major challenge for future research. Redundancies and specificities in the ability of many protein kinases to sense and signal both biotic and abiotic stresses represent a fascinating challenge to decipher the key players that transform the activated combinatorial networks to a precise response.

## Author Contributions

GR-H and MM conceived and contributed to the final version of the manuscript.

## Conflict of Interest

The authors declare that the research was conducted in the absence of any commercial or financial relationships that could be construed as a potential conflict of interest.

## Publisher’s Note

All claims expressed in this article are solely those of the authors and do not necessarily represent those of their affiliated organizations, or those of the publisher, the editors and the reviewers. Any product that may be evaluated in this article, or claim that may be made by its manufacturer, is not guaranteed or endorsed by the publisher.

## Footnotes

1
http://www.proteomexchange.org

2
https://phosphat.uni-hohenheim.de/
